# Effect of Pre-Oxidation on High-Temperature Oxidation Behavior of Al-Si Coating on Nickel-Based Superalloy

**DOI:** 10.3390/ma15217440

**Published:** 2022-10-23

**Authors:** Yanmei Li, Haishuang Lv, Yabin Li, Naiwen Fan

**Affiliations:** The State Key Laboratory of Rolling and Automation, Northeastern University, Shenyang 110819, China

**Keywords:** Al-Si coating, pre-oxidation, high-temperature oxidation, oxide film

## Abstract

Under the condition of long-time high-temperature oxidation, oxidation in Al-Si coating will lead to degradation of the coating. To solve this problem, the Al-Si coating was treated before the oxidation experiment at different pre-oxidation temperatures and times. The structure, morphology and element distribution of the oxide film were characterized by XRD, SEM and EPMA, and the oxidation kinetics curves were drawn. The results show that the oxidation resistance of the pre-oxidized samples is improved in the process of constant temperature oxidation at 1000 °C. When the pre-oxidation temperature is set at 950 °C, the mass gain of the samples is the lowest, and the accelerated decomposition of Cr_2_O_3_ caused by high pre-oxidation temperature is avoided. After oxidation for 300 h, the oxide film on the surface is still continuous and dense without peeling. The samples pre-oxidized at 950 °C for 7 h show the best oxidation resistance in a 1000 °C constant temperature oxidation process, more α-Al_2_O_3_ oxides are generated on the coating surface and the element diffusion between the matrix and the coating caused by overly long oxidation time is avoided.

## 1. Introduction

Superalloys have been widely used in aerospace hot end components because of their excellent high-temperature oxidation resistance, heat corrosion resistance, fatigue performance and high-temperature mechanical properties. With the development of turbine engine technology, its working temperature is required to be higher and higher. The temperature in front of the turbine of an F119 fourth-generation military engine in the United States has reached 1676 °C, and the lowest temperature in front of the turbine of major foreign military engines is about 1266 °C [1,2]. At present, the maximum temperature that a nickel-based single-crystal superalloy can withstand has only reached 1330 °C [3]. In addition, the mechanical properties and oxidation resistance of the alloy will be greatly reduced due to the interaction between high temperature and gas. Although the mechanical properties of the alloy can be improved by adding some solid-solution-strengthened alloy elements (W, Mo, V, etc.), at the same time, the content of elements with anti-oxidation and anti-corrosion effects (Al, Cr, etc.) in the alloy will be reduced, so it cannot meet the high-temperature mechanical properties and high-temperature oxidation resistance of the material at the same time. Generally, improvement in oxidation resistance of alloys is mainly achieved by alloying and surface modification. Improvement in the oxidation resistance and corrosion resistance of superalloys is mainly achieved by surface coating in addition to adding Cr elements [4,5]. Adding high-temperature protective coating on the surface of a superalloy can not only significantly improve its high-temperature oxidation and corrosion resistance but also reduce the production cost; this has been widely used in turbine engine blades. Among them, Al-Si coating has a simple process and low cost and is the only high-temperature protection coating that can resist type I and type II hot corrosion. With the improvement in engine technology, there are higher requirements for better protective performance of coatings, not only to ensure good oxidation resistance at high temperature but also to reduce the huge economic costs caused by frequent workpiece replacement and coating preparation. At present, although different processes are used to improve the protective performance of the coating, the repreparation process of the coating is relatively complex, and the cost of preparing the coating will be further increased. Therefore, some researchers found that the pre-oxidation process can effectively improve the high-temperature oxidation resistance of the coating without changing the structure of the coating, and the pre-oxidation process has a low cost and simple operation, making it an effective means to improve the protective performance of the coating. A layer of oxide film is formed on the coating surface after pre-oxidation treatment, which prevents the external O^2−^ from contacting the coating surface directly and reduces the internal oxidation rate of the coating.

The pre-oxidation process is widely used for metals and superalloys. The main purpose of this process is to pre-oxidize the material at a certain temperature and time to form a protective oxide film on the surface of the material. This oxide film can slow down the subsequent oxidation rate of the material and improve the high-temperature oxidation resistance of the material. In recent years, pre-oxidation technology has received extensive attention due to its simple operation and low cost [6,7,8]. The pre-oxidation process is also applicable to high-temperature protective coating. In recent years, through studying the influence of pre-oxidation on the high-temperature oxidation and heat resistance corrosion properties of the coating, Yang [9] found that a Pt-Al-coated sample can form a continuous and dense Al_2_O_3_ film after pre-oxidation treatment, which can effectively prevent molten salt from directly entering the material surface, and the pre-oxidized samples did not indicate internal oxidation or a vulcanization phenomenon. In the process of a hot corrosion experiment, the oxide film formed on the surface of the pre-oxidized Al-Si coating is thin and maintains good integrity, but the structure of the oxide film formed fluctuates greatly, indicating that the oxide film grows toward the inner layer of the coating during the process of oxidation dissolution [10,11]. Gao Bo et al. [12] studied the influence of oxidation on the high-temperature oxidation and hot corrosion behavior of Co-Al-W-based superalloy and found that, when the alloy pre-oxidized at 950 °C under normal oxygen, partial pressure was oxidized at 1000 °C, the oxidation products fell off less, the oxidation mass gain was slowed down and the oxidation resistance of the alloy was improved. Bai Yin et al. [13] studied the effect of pre-oxidation treatment on the oxidation behavior of G115 steel at high-temperature vapor and found that the pre-oxidation process could not only improve the oxidation resistance of G115 steel in the short-term but also reduce the oxidation mass gain by about 30% on the whole. Tu Jiangping et al. [14] studied the corrosion behavior of Ni-Cr-Fe alloy in high-temperature chlorine gas after air pre-oxidation at 700 °C. Taniguchi et al. [15] studied the improvement in the antioxidant properties of Al films deposited on Fe-Cr-Al sheets by pre-oxidation. Chen et al. [16] pre-oxidized the double-layer thermal barrier coating with atmospheric plasma spraying Co-32Ni-21Cr-8A1-0.5Y coating as the bonding layer and found that pre-oxidation at low pressure could promote formation of a dense layer of A1_2_O_3_ between the metal bonding layer and ceramic thermal barrier layer and inhibit generation of other harmful oxides. Thus, the high-temperature oxidation resistance of the coating is significantly improved.

Pre-oxidation can significantly improve the thermal corrosion resistance of the coating, and there are many studies in this area. The reported coating type is mainly thermal barrier coating, which can inhibit formation of spinel oxide after pre-oxidation treatment, and the oxides formed by the thermally grown oxide (TGO) layer are mainly α-Al_2_O_3_ [17]. Mo et al. [18] studied the effect of pre-oxidation on the hot corrosion resistance of an aluminum siliconized layer and found that pre-oxidation treatment increased the hot corrosion resistance considerably of a series of ASL-3 type Al-Si coatings but did not damage any mechanical properties of the coating/substrate system. Xu et al. [19] studied the thermal corrosion behavior of pre-alumina silicon coating in mixed sulfate at 1150 °C and found that pre-oxidation treatment could improve the thermal corrosion resistance of Al-Si coating at more than 1000 °C, and the thermal corrosion kinetics curve showed an approximately parabolic law in the first 60 h. Ning et al. [20] studied the effect of vacuum pre-oxidation on the microstructure and hot corrosion performance of cold-sprayed CoNiCrAlY coating. They found that vacuum pre-oxidation treatment can improve the bonding between coating particles, make the coating denser and generate a continuous and dense α-Al_2_O_3_ oxide film with a thickness of about 0.26 μm on the coating surface, which effectively slows down the diffusion rate of elements, such as S and Cl, into the coating, and improves the corrosion resistance of the coating by nearly twice. For the Al-Si coating, the research mainly focuses on the influence of pre-oxidation on the thermal corrosion resistance of the Al-Si coating, and the research on the high-temperature oxidation resistance of the Al-Si coating is less prominent. In addition, the research on the influence of pre-oxidation on the high-temperature oxidation resistance of the Al-Si coating is just a simple study of the role of pre-oxidation through pre-oxidation treatment, and there is no systematic study on the influence of pre-oxidation process parameters on the high-temperature oxidation resistance and thermal corrosion resistance of the Al-Si coating. That is, there are few reports on the influence of pre-oxidation temperature and time on the high-temperature oxidation resistance of Al-Si coating. In this paper, the Al-Si coating is taken as the benchmark sample, and, through the pre-oxidation treatment of the Al-Si coating, the influence of the pre-oxidation process on the high-temperature oxidation resistance of the Al-Si coating is studied, the reasonable pre-oxidation process parameters are determined and the influence of pre-oxidation on the high-temperature oxidation behavior of the Al-Si coating is analyzed.

## 2. Materials and Methods

**Figure 1:**
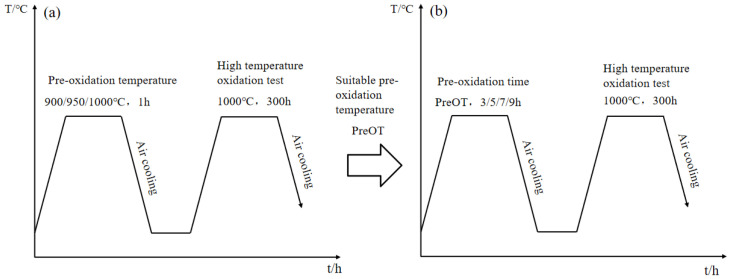
Experimental scheme of pre-oxidation process under high-temperature oxidation: (**a**) different pre-oxidation temperatures; (**b**) different pre-oxidation times.

In this paper, DZ417G nickel-based superalloy (see Table 1 for chemical composition) was used as the matrix material, and Al-Si coating was prepared by slurry method. First, the ball abrasive slurry was carried out. The slurry was mainly composed of infiltration agent and binder. The NiAl alloy powder and Si powder were mixed in a certain proportion, NH_4_Cl of 1% total mass was added to it and the infiltration agent was created after ball milling and roasting. At the same time, the ethanol solution was diluted to a certain proportion of polyvinyl alcohol and water to create binder. Sand-blowing, spraying, aluminizing silicon and vacuum diffusion were then carried out, and, finally, shot peening was performed. Before the constant temperature oxidation experiment at 1000 °C, the samples were pre-oxidized at different temperatures and times, as shown in Figure 1. The specific experimental steps were as follows:

(1) Pre-oxidation temperature

After the sample had been cleaned and dried, it was cooled to room temperature and then stored in the drying dish. Then, the samples were put into the crucible fired to constant weight and held at 900 °C, 950 °C and 1000 °C for 1 h, respectively. The samples were taken out and cooled to room temperature and then weighed on a precision electronic balance. The average value of three times of weighing was taken as the initial weight before constant temperature oxidation. Three sets of parallel specimens were set for each temperature. After pre-oxidation treatment, the crucible containing the sample was put into the SXL-1400 °C box-type resistance furnace heated to 1000 °C. After holding for 10 h, 25 h, 50 h, 100 h, 150 h, 200 h, 250 h and 300 h, the sample was removed for cooling and weighing and then put back into the furnace until next weighing. The suitable pre-oxidation temperature (PreOT) was obtained by drawing the oxidation kinetics curves and analyzing the surface oxidation morphology and coating structure.

(2) Pre-oxidation time

The pre-oxidation time was set to 3 h, 5 h, 7 h and 9 h, where the pre-oxidation temperature T is the PreOT obtained earlier. The experimental method was similar to the previous one. Before the constant temperature oxidation experiment, it was required to pre-oxidize at temperature T for 3 h, 5 h, 7 h and 9 h, respectively, and then conduct the constant temperature oxidation experiment at 1000 °C for 300 h. The isothermal oxidation kinetics curves were plotted using OriginPro 2017C version b9.4.2.380, OriginLab Corporation, Northampton, NC, USA to obtain a suitable pre-oxidation time.

The oxidized sample was cut in half by wire cutting, and the sample was pre-ground and corroded after thermal inlaying. Smartlab9KW X-ray diffraction was used to analyze the phase of the oxidation products on the surface of the sample. Zeiss Ultra 55 scanning electron microscope [21] was used to observe the oxidation morphology and cross-section morphology of the sample. JEOL-JXA-8530F field emission electron probe was used to observe the distribution of elements on the cross-section of the coating.

## 3. Results

### 3.1. Microstructure of Coatings at Different Pre-Oxidation Temperatures

**Figure 2:**
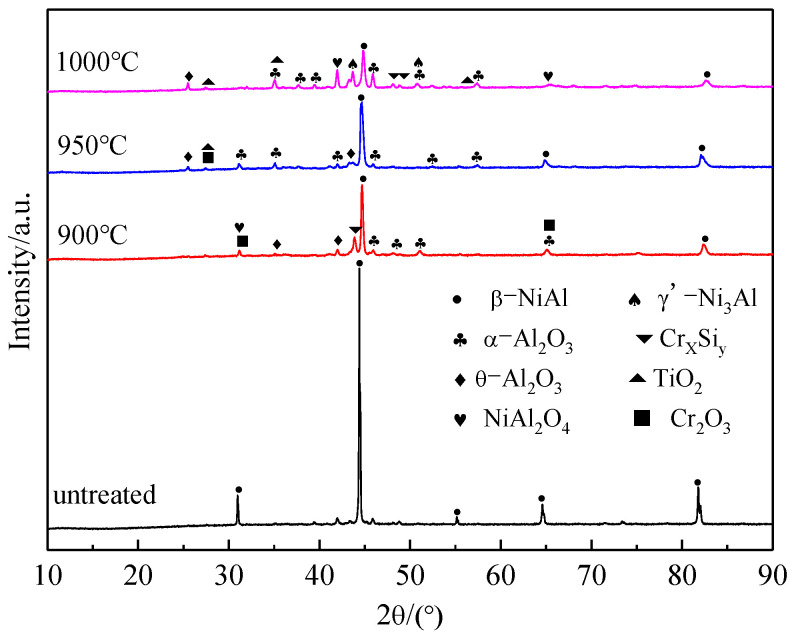
XRD patterns of Al-Si coatings pre-oxidized for 1 h at different pre-oxidation temperatures.

As shown in Figure 2, the surface of Al-Si coating without pre-oxidation treatment is a single β-NiAl phase. Besides the β-NiAl phase, α-Al_2_O_3_, θ-Al_2_O_3_ and Cr_2_O_3_ oxides are also generated on the surface of the Al-Si coating sample after pre-oxidation treatment. With the increase in pre-oxidation temperature, the number of diffraction peaks of the Cr_2_O_3_ phase gradually decreases, while that of TiO_2_ gradually increases. The diffraction peak of Cr_2_O_3_ almost disappears at 1000 °C. It is reported [22,23] that Cr_2_O_3_ is easy to be oxidized to form volatile CrO_3_ when oxidized at 1000 °C, which damages the integrity of the alloy oxide layer. With the increase in the pre-oxidation temperature, the mutual diffusion rate of elements between the coating and the matrix is also accelerated, and the decomposition of Cr_2_O_3_ is more rapid. Thus, the oxide formed on the coating surface is mainly Al_2_O_3_. In addition, as both Ti and Al are elements with strong oxygen affinity, in the selective oxidation process, Ti in the matrix diffuses rapidly and easily forms TiO_2_ above Al_2_O_3_ on the surface, which can also improve the growth rate of the oxide film. At the same time, Ti in the oxide film can promote the transformation of metastable θ-Al_2_O_3_ to stable α-Al_2_O_3_ [24,25]. This can ensure that the surface oxide film is mainly composed of stable α-Al_2_O_3_ and improve its high-temperature oxidation resistance. However, although this transformation can reduce the growth stress in the growth process of Al_2_O_3_, it will increase its hot compression stress and lead to coating peeling or cracking [26]. A small amount of spinel oxide NiAl_2_O_4_ was also formed at 950 °C and 1000 °C. When the pre-oxidation temperature is 950 °C, the number of diffraction peaks of α-Al_2_O_3_ is the largest, which indicates that the transition of metastable oxide θ-Al_2_O_3_ to stable α-Al_2_O_3_ oxide is promoted at this temperature.

**Figure 3:**
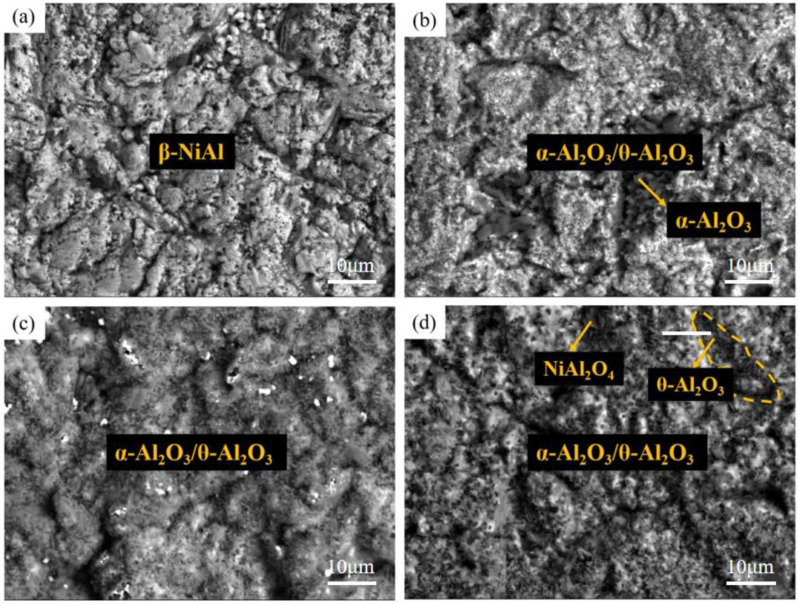
The surface morphologies of the coating after pre-oxidation at different temperatures for 1 h: (**a**) untreated; (**b**) 900 °C; (**c**) 950 °C; (**d**) 1000 °C.

According to Figure 3, there is a continuous oxide film mainly composed of α-Al_2_O_3_ and θ-Al_2_O_3_ on the surface of the pre-oxidized samples. The surface of PreO-900 °C is flatter, with an obvious black bulk area, which is mainly Al oxide and contains a small amount of Ti. The oxide films on the surface of PreO-950 °C and PreO-1000 °C are continuous and dense, which are composed of clusters of oxides, mainly α-Al_2_O_3_. At PreO-1000 °C, some of the clustered oxides on the surface of the Al-Si coating are needle-like oxides, which can be identified as spinel Ni(Cr,Al)_2_O_4_ based on shape analysis. This oxide has poor oxidation resistance and is easy to flake off. In the three groups of pre-oxidized samples, the oxide film on the surface of pre-oxidized samples at preo-950 °C is more continuous and dense, mainly α-Al_2_O_3_, and only a small amount of acicular oxides.

**Figure 4:**
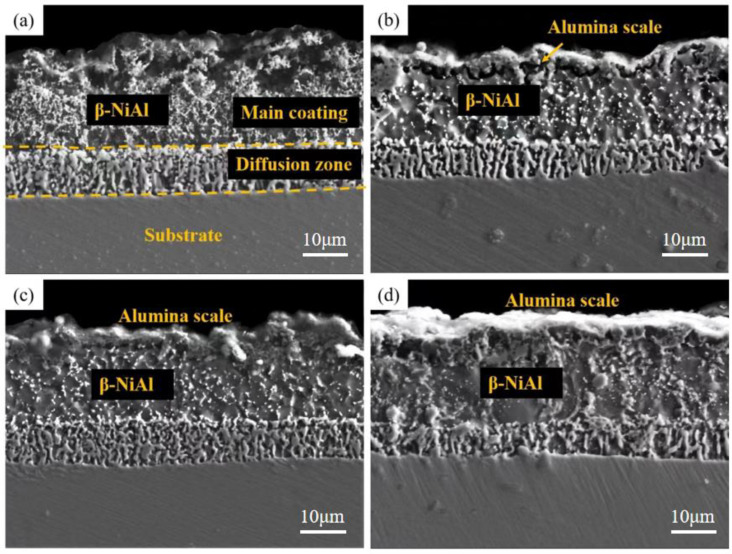
Cross-sectional morphologies of Al-Si coatings after pre-oxidation at different pre-oxidation temperatures for 1 h: (**a**) untreated; (**b**) 900 °C; (**c**) 950 °C; (**d**) 1000 °C.

**Figure 5:**
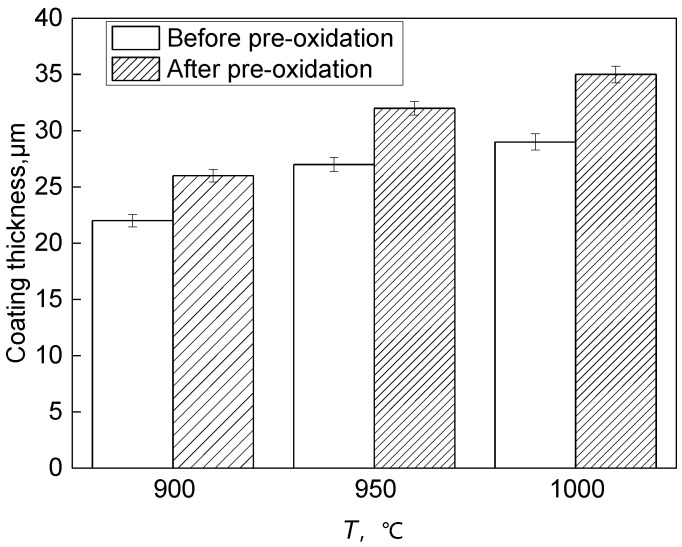
Thickness of Al-Si coating after pre-oxidation at different pre-oxidation temperature for 1 h.

**Figure 6:**
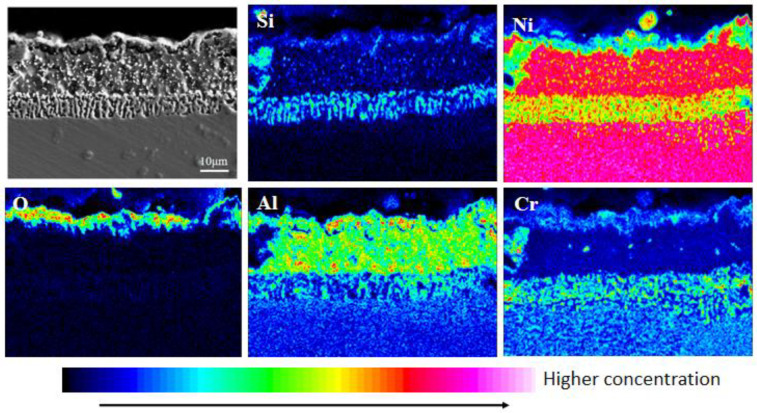
Element distribution of coating section after pre-oxidation at 900 °C for 1 h.

According to Figure 4 and Figure 5, the outermost layer of the Al-Si coating after pre-oxidation for 1 h has a continuous oxide film. The diffusion layer and the main part of the coating do not change significantly, and the coating thickness increases. After 1 h of pre-oxidation treatment, a certain thickness of oxide film is formed on the outer layer of the coating, and short-term pre-oxidation treatment will not affect the coating structure. Figure 6 shows the element distribution after pre-oxidation at 900 °C for 1 h. The outer layer of the coating is mainly composed of the oxide layer and Al-rich β-NiAl phase, wherein the oxide layer is mainly oxides of α-Al_2_O_3_ and θ-Al_2_O_3_. Moreover, according to the distribution of Al and O elements shown in Figure 6, the Al_2_O_3_ oxide film formed is continuous, indicating that ‘pre-oxidation treatment at a certain temperature can promote formation of protective oxide film. In pre-oxidized coating, the distribution of Si is low on the outside and high on the inside, indicating that, during the high-temperature oxidation process, Si will diffuse towards the matrix and combine with the refractory metal elements, such as Mo and Co, diffused outward from the matrix to form Si-rich M_6_C (M stands for the general term of carbide-forming elements, and M6C is the general term of this class of carbides containing one carbon atom and six alloying element atoms in a carbide lattice). The pre-oxidation treatment can further improve the promotion effect of Si on formation of M_6_C and reduce the interdiffusion of elements between the coating and the substrate in the subsequent oxidation experiments [27,28], and the formed oxide film can also prevent direct contact of external oxygen with the coating surface.

**Figure 7:**
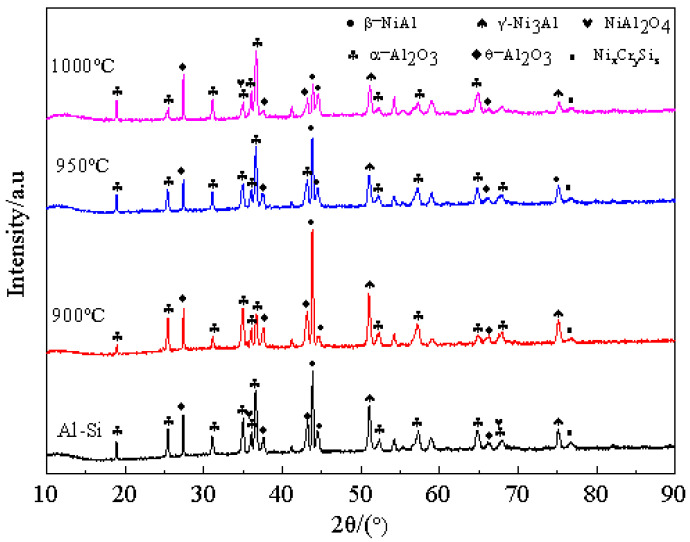
XRD patterns of Al-Si coatings at different pre-oxidation temperatures after constant temperature oxidation at 1000 °C for 300 h.

As shown in Figure 7, when the coating is pre-oxidized at 1000 °C for 300 h, the number of α-Al_2_O_3_ diffraction peaks formed on the coating surface is less than that of the other three samples, and the amount of γ′-Ni_3_Al is more, indicating that the coating degrades after 300 h of oxidation. That is, the interdiffusion of elements and the large consumption of Al elements in the oxide film occur in the oxidation process, resulting in the transformation of part of β-NiAl into γ ’-Ni_3_Al, which leads to the reduction of high temperature oxidation resistance of the coating. However, the number of γ′-Ni_3_Al diffraction peaks of the samples treated at 900 °C and 950 °C pre-oxidation temperature is less than that of the samples treated with untreated Al-Si coating and 1000 °C pre-oxidation temperature. The samples pre-oxidized at 950 °C for 300 h have the largest number of β-NiAl and α-Al_2_O_3_ diffraction peaks and only a few weak θ-Al_2_O_3_ diffraction peaks. The presence of β-NiAl can ensure that the Al-Si coating can still form a continuous Al_2_O_3_ oxide film on the surface of the coating under a long duration of high-temperature oxidation, which also indicates that the pre-oxidation treatment at 950 °C can not only promote the transformation of θ-Al_2_O_3_ to α-Al_2_O_3_ but also ensure the stability of the surface oxide film. This can greatly improve the high-temperature oxidation resistance of the coating.

**Figure 8:**
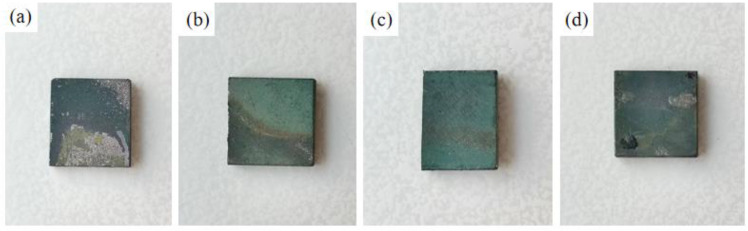
Macroscopic morphologies of coatings under different pre-oxidation temperatures at 1000 °C for 300 h: (**a**) untreated; (**b**) 900 °C; (**c**) 950 °C; (**d**) 1000 °C.

**Figure 9:**
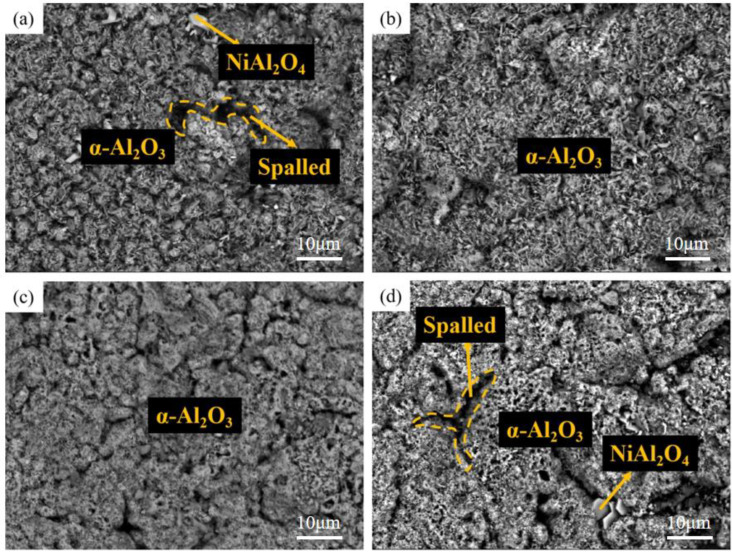
Oxidation morphologies of coatings under different pre-oxidation temperatures at 1000 °C for 300 h: (**a**) untreated; (**b**) 900 °C; (**c**) 950 °C; (**d**) 1000 °C.

**Figure 10:**
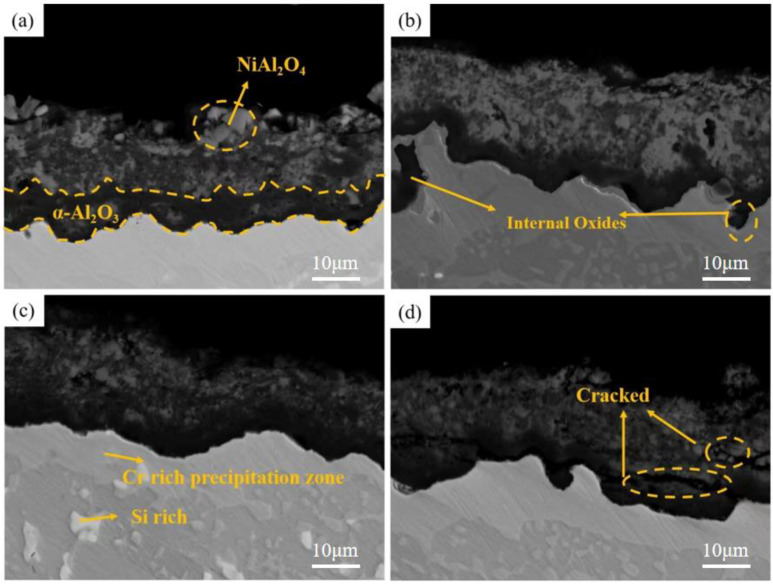
Cross-sectional morphologies of coatings after constant temperature oxidation at 1000 °C for 300 h at different pre-oxidation temperatures: (**a**) untreated; (**b**) 900 °C; (**c**) 950 °C; (**d**) 1000 °C.

**Figure 11:**
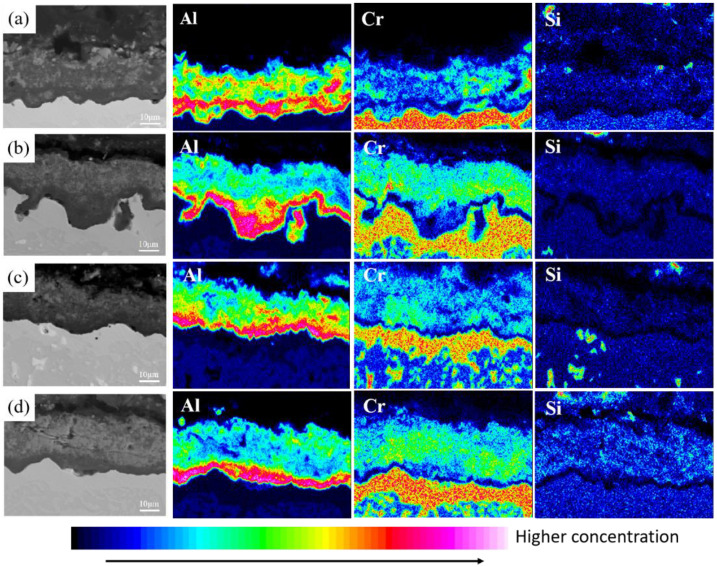
Element distribution of Al-Si coating at different pre-oxidation temperatures at 1000 °C constant temperature oxidation for 300 h: (**a**) untreated; (**b**) 900 °C; (**c**) 950 °C; (**d**) 1000 °C.

Figure 8, Figure 9 and Figure 10 show the macroscopic, surface and cross-sectional morphologies of the coating after pre-oxidation at different temperatures and then constant-temperature oxidation at 1000 °C for 300 h, respectively. The surface of the sample pre-oxidized at 950 °C is the most smooth and flat, followed by that at 900 °C. The surfaces of the sample pre-oxidized at 1000 °C and un-pre-oxidized are uneven. The surfaces of the four groups of samples are mainly relatively continuously ridged α-Al_2_O_3_, and there are obvious NiAl_2_O_4_ spinel oxides on the outside of the non-pre-oxidized coating. The oxide film in contact with the outside world is broken. However, according to the element distribution shown in Figure 11, the distribution of Al is still continuous, indicating that it still has good resistance to high-temperature oxidation. The coating pretreated at 900 °C has an internal oxidation phenomenon, which is due to further reaction of Cr_2_O_3_ generated during oxidation with oxygen to generate volatile CrO_3_, resulting in thinner oxide film so that O can enter the coating more easily. The surface oxide film of Al-Si coating with a pre-oxidation temperature of 950 °C is relatively continuous and dense, and no obvious peeling phenomenon is found, while the surfaces of the other three groups of coatings demonstrate a slight peeling phenomenon. EDS analysis at the exfoliation site shows that the contents of Al and O are still relatively high, indicating that the new Al_2_O_3_ film is still formed there, which also indicates that the oxidation process of Al-Si coating is accompanied by exfoliation and regeneration of the oxide film. With the growth in oxidation time, before the oxidation film is destroyed, the Al source in the coating combines with the outside O in time to form a new oxide film through diffusion so as to ensure that the coating has long-term high-temperature oxidation resistance. Under pre-oxidation treatment at 1000 °C, a part of NiCr_2_O_4_ spinel oxide was also formed on the surface of the coating, and the cracking phenomenon occurred in the oxidation film area inside the coating. From the XRD pattern, it can be seen that the transformation of β-NiAl to γ′-Ni_3_Al at this temperature accelerated the consumption of Al in the coating, and the protective oxide Al_2_O_3_ could not be fully formed. Therefore, the oxide film will crack under high-temperature oxidation for a long duration.

### 3.2. Microstructure of Coatings at Different Pre-Oxidation Times

**Figure 12:**
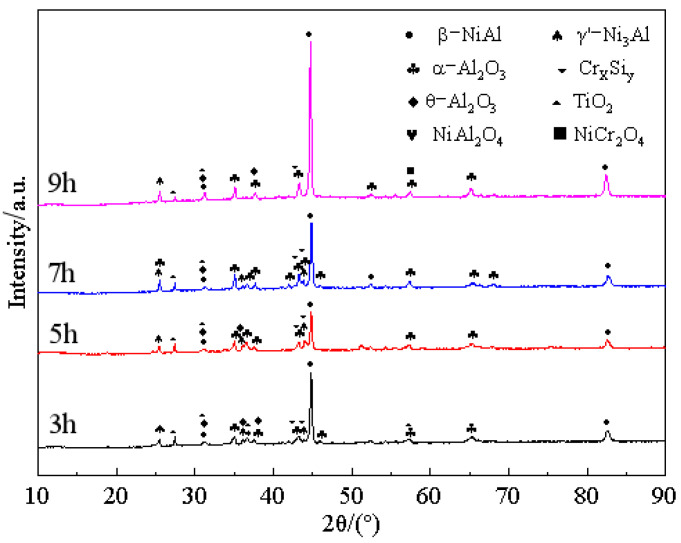
XRD patterns of Al-Si coating surface with different pre-oxidation times at 950 °C.

The most appropriate pre-oxidation temperature has been determined as 950 °C by previous analysis. The influence of pre-oxidation time on the oxidation performance of the coating is studied below. Figure 12 shows the XRD patterns of the coating surface treated with different pre-oxidation times at 950 °C. After 3 h, 5 h, 7 h and 9 h pre-oxidation treatment, relatively stable oxide α-Al_2_O_3_ is formed on the surface of the coating, but a strong β-NiAl diffraction peak and part of the γ′-Ni_3_Al phase also appear, indicating that the coating degrades during the oxidation stage. When the pre-oxidation time is 9 h, a small amount of Ni(Al,Cr)_2_O_4_ spinel oxide is produced on the surface of the sample. As can be seen from Figure 12, with the increase in pre-oxidation time, the number of TiO_2_ diffraction peaks decreases, while the number of α-Al_2_O_3_ diffraction peaks increases, which indicates that prolonging the pre-oxidation time within a certain range is helpful to improve the high-temperature oxidation resistance of Al-Si coating.

**Figure 13:**
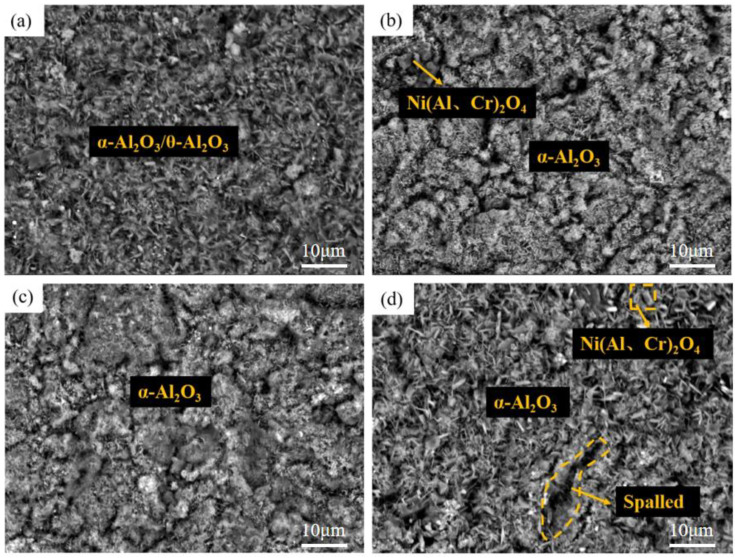
Surface morphologies of Al-Si coatings with different pre-oxidation times at 950 °C: (**a**) 3 h; (**b**) 5 h; (**c**) 7 h; (**d**) 9 h.

**Figure 14:**
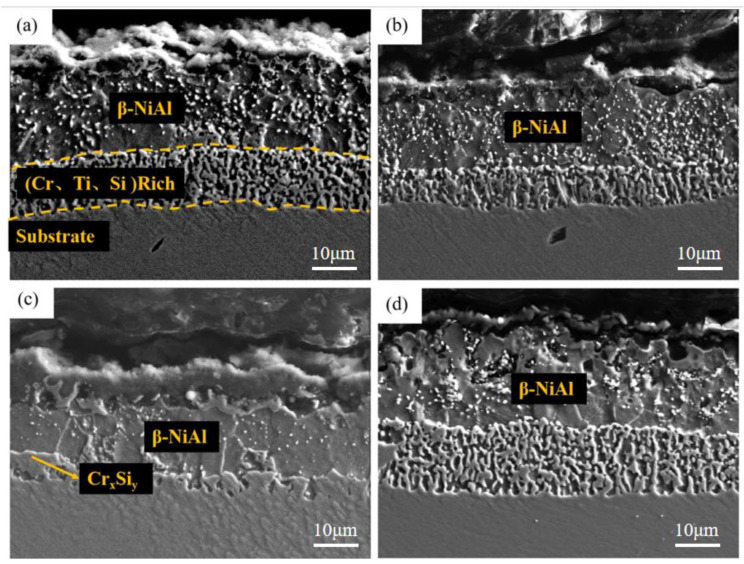
Cross-sectional morphologies of Al-Si coatings with different pre-oxidation times: (**a**) 3 h; (**b**) 5 h; (**c**) 7 h; (**d**) 9 h.

Figure 13 and Figure 14 show the surface and cross-section morphology of Al-Si coating treated with different pre-oxidation times. The surface of the four groups of samples is relatively flat, and the outermost layer of continuous oxide film is mainly composed of lamellar α-Al_2_O_3_ and some needle-like θ-Al_2_O_3_, and the mutual diffusion zone is mainly β-NiAl phase. A pre-oxidation time of 3 h ensures that the protective oxide film is formed on the coating surface in advance. As can be seen from Figure 13a, there are more needle-like oxides on the surface of the oxide film, and these needle-like oxides are easy to form gaps, which provides a diffusion channel for entry of external O^2−^, thus accelerating the consumption of Al in the coating, which is not conducive to subsequent long-term resistance to high-temperature oxidation. When the pre-oxidation time is 7 h, the oxide film on the coating surface is mainly α-Al_2_O_3_, and the oxide film is mainly lamellar. The surface is more compact, with no spinel oxide. At the same time, the particle γf′-Ni_3_Al precipitated in the interdiffusion region decreased. When the pre-oxidation time is 9 h, the oxide film on the surface of the coating is relatively intact, but there are some faults in the middle, and there are some gaps between it and the interdiffusion zone. It can be seen from Figure 13d that, when the pre-oxidation time is 9 h, the oxide on the coating surface is mainly lamellar, while the amount of oxide in the form of needle tip decreases and Ni(Al,Cr)_2_O_4_ spinel oxide appears. At the same time, Figure 13d shows that the granular γ′-Ni_3_Al precipitated in the mutual diffusion zone slightly increases, and formation of γ′-Ni_3_Al and surface spinel oxide indicates that the oxide film appears to exfoliate.

**Figure 15:**
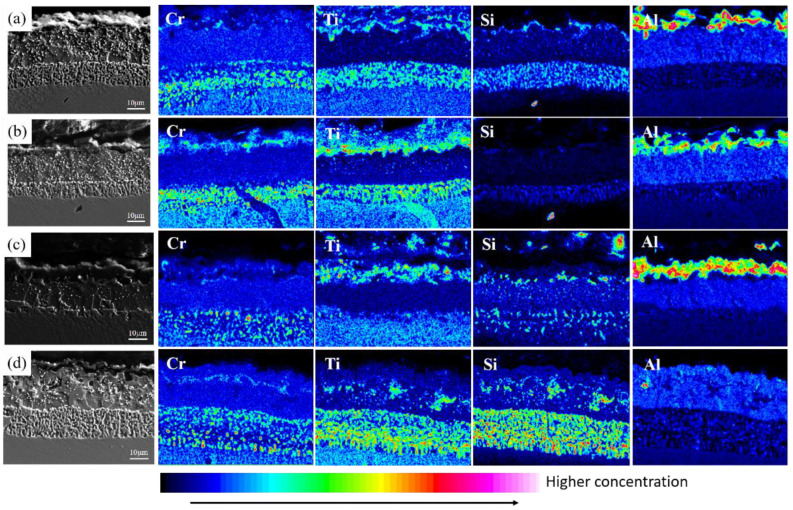
Element distribution of Al-Si coating section with different pre-oxidation times: (**a**) 3 h; (**b**) 5 h; (**c**) 7 h; (**d**) 9 h.

After pretreatment at different pre-oxidation temperatures, Cr and Si are mainly distributed in the diffusion zone below the diffusion layer and combine with refractory particles (Mo, V and other elements) in the matrix to form M_6_C, which plays a certain diffusion barrier role and hinders mutual diffusion of some elements between the matrix and the coating. This rule is also observed at different pre-oxidation times. Figure 15 shows the distribution of cross-section elements of the coating at different pre-oxidation times at 950 °C. Besides comparing the distribution of Cr and Si elements, the distribution of Ti in the coating was also observed. TiO_2_ is attached to the Al_2_O_3_ oxide film, and it is found through the distribution of cross-section elements that Ti is mainly distributed in the oxide layer and diffusion zone under the pre-oxidation treatment. In the pre-oxidation process, Ti has a strong affinity with O, and, when the temperature exceeds 800 °C, the oxidation behavior of rupture will occur. TiO_2_ formed in the oxide film will easily penetrate the coating, form a rapid diffusion channel, destroy the compactness of the oxide film and accelerate degradation of the coating.

**Figure 16:**
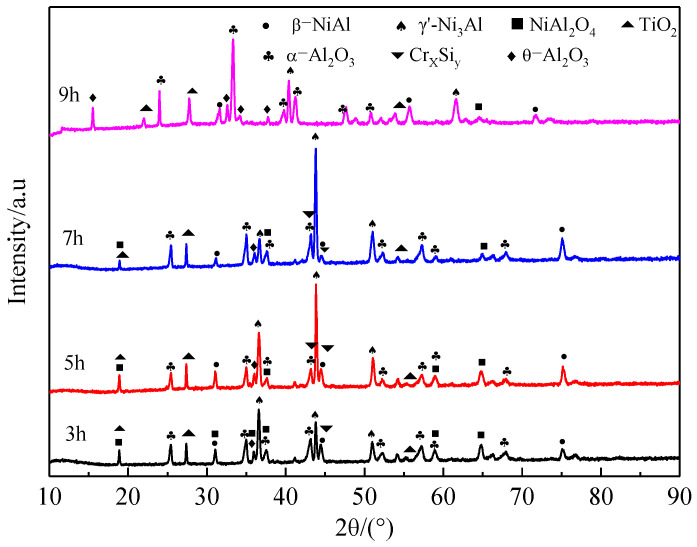
XRD patterns of Al-Si coatings with different pre-oxidation times at 1000 °C for 300 h.

Figure 16 shows the XRD patterns of Al-Si coating oxidized at 1000 °C for 300 h at different pre-oxidation times. The number of diffraction peaks of the γ′-Ni_3_Al phase on the coating surface increases compared with that at 10 h oxidation, which indicates that the oxidation film spalling is more serious at this time. At the same time, the TiO_2_ generated on the coating surface after pre-oxidation treatment is still too much, which is not conducive to long-term oxidation of the coating. After oxidation for 300 h, the diffraction peaks of θ-Al_2_O_3_ and spinel oxide in the coating treated with pre-oxidation for 7 h are less, while the diffraction peaks of α-Al_2_O_3_ and β-NiAl are more. It can be concluded that the 7 h pre-oxidation treatment is conducive to formation of a more continuous and compact α-Al_2_O_3_ oxide film on the coating surface, which promotes transformation of θ-Al_2_O_3_ to α-Al_2_O_3_ in the early oxidation stage.

**Figure 17:**
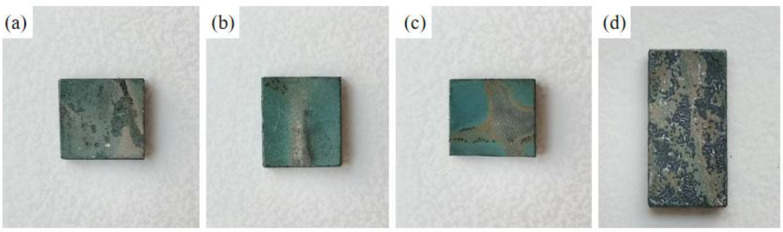
Macroscopic morphologies of Al-Si coatings oxidized at 1000 °C for 300 h under different pre-oxidation times: (**a**) 3 h; (**b**) 5 h; (**c**) 7 h; (**d**) 9 h.

**Figure 18:**
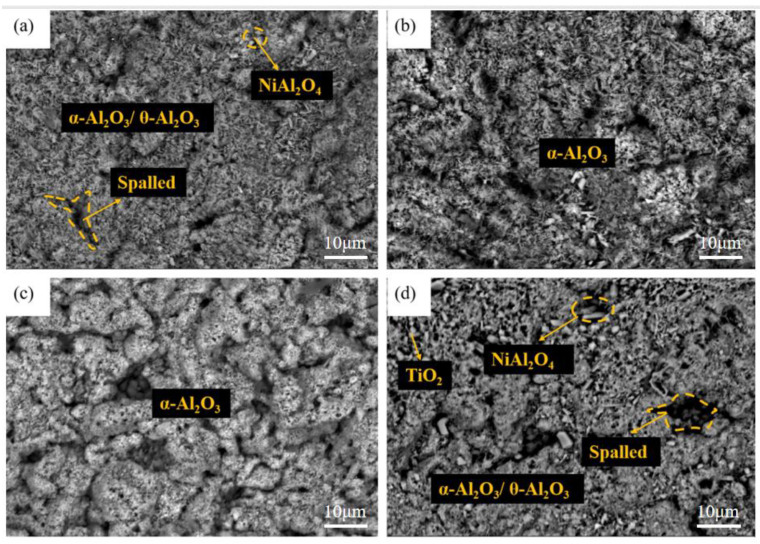
Surface morphologies of Al-Si coatings oxidized at 1000 °C for 300 h under different pre-oxidation times: (**a**) 3 h; (**b**) 5 h; (**c**) 7 h; (**d**) 9 h.

**Figure 19:**
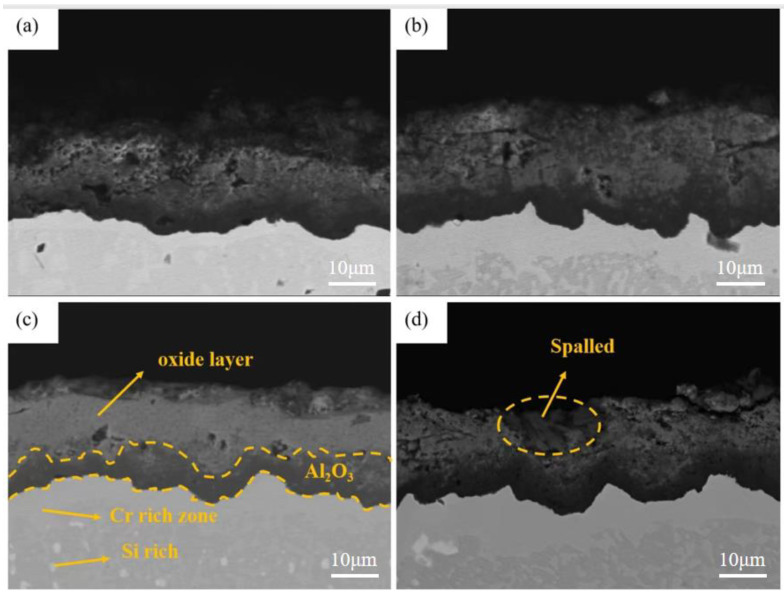
Cross-sectional morphologies of Al-Si coatings oxidized at 1000 °C for 300 h under different pre-oxidation times: (**a**) 3 h; (**b**) 5 h; (**c**) 7 h; (**d**) 9 h.

Figure 17, Figure 18 and Figure 19 show the macroscopic, surface and cross-sectional morphologies of the coating after pre-oxidation at different temperatures and then constant-temperature oxidation at 1000 °C for 300 h, respectively. The surface effect of samples pre-oxidized at 950 °C for 5 h and 7 h is better than that of samples pre-oxidized for 3 h and 9 h. The surface of the four groups of samples is relatively flat, and the oxide film is relatively complete and continuous. The outer oxide layer is mainly composed of Cr, Ti and Al oxides, and the black structure is mainly α-Al_2_O_3_. There is a Cr-rich precipitation zone below the α-Al_2_O_3_ layer, and a Si-rich M_6_C precipitation zone is formed between the matrix and the mutual diffusion zone. The sample surface with pre-oxidation time of 3 h and 9 h has different degrees of oxide film peeling. Due to the thermal stress of the oxide film growth, oxide film peeling occurs at this place, and the oxidation rate is accelerated. According to EDS, the peeling place is mainly θ-Al_2_O_3_. Although θ-Al_2_O_3_ is an oxide with poor oxidation resistance, it still has some oxidation resistance.

**Figure 20:**
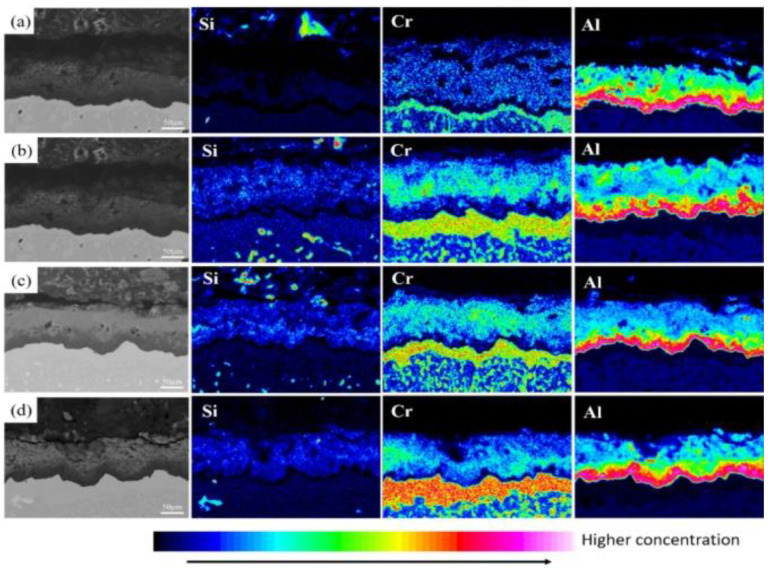
Element distribution of Al-Si coating at 1000 °C constant temperature oxidation for 300 h with different pre-oxidation times: (**a**) 3 h; (**b**) 5 h; (**c**) 7 h; (**d**) 9 h.

At different pre-oxidation times, Figure 15 shows that Ti is mainly distributed in the outer layer of the coating and the precipitation zone rich in Cr, Si and other elements during pre-oxidation. Formation of more TiO_2_ was found when the oxidation time was 300 h. Figure 18d and Figure 19d show that long strips of TiO_2_ and typical spinel oxide Ni(Al,Cr)_2_O_4_ are generated on the surface of the coating after 9 h of pre-oxidation, and the outer layer of the oxide film is broken. Meanwhile, it can be observed in Figure 20 that, with the increase in pre-oxidation time, the content of Al element in the outer oxide is relatively reduced, which will lead to formation of spinel oxide, which is not conducive to the oxidation resistance of the coating. Although increasing the pre-oxidation time can improve the high-temperature oxidation resistance of the coating to a certain extent, the longer the pre-oxidation time, the faster the interdiffusion of elements between the coating and the matrix, which is not conducive to improvement in the performance. After 300 h oxidation of the samples with a pre-oxidation time of 7 h, although there was also peeling on the coating surface, it was less than that of the other three groups of samples. Moreover, the oxide film on the surface was flatter, mainly lamellar, dominated by α-Al_2_O_3_ and no obvious long strip oxide was found, which is consistent with the oxidation trend of the previous oxidation kinetic curve. At the same time, Al and other antioxidant elements were still abundant in the coating, which plays an important role in improving the oxidation resistance of the coating. In conclusion, the high-temperature oxidation resistance of the coating is best when it is pre-oxidized for 7 h.

## 4. Discussion

### 4.1. Effect of Pre-Oxidation Temperature on High-Temperature Oxidation Behavior of Al-Si Coating

**Figure 21:**
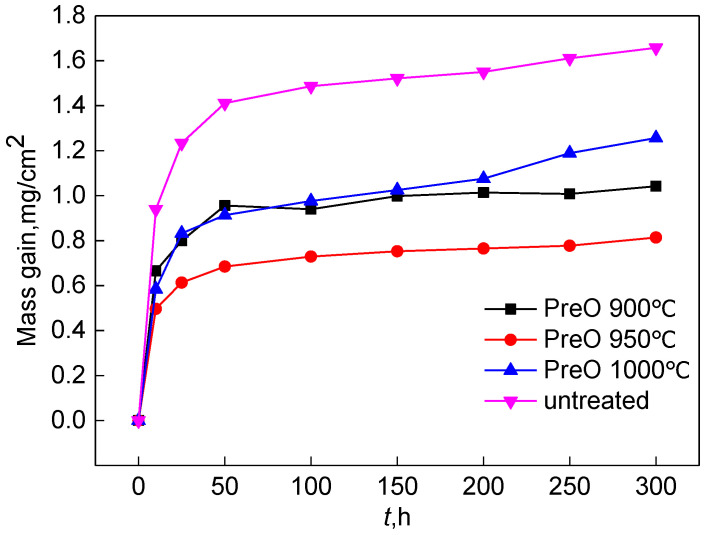
Oxidation kinetics curves of Al-Si coatings at different pre-oxidation temperatures at 1000 °C.

Figure 21 shows the constant temperature oxidation kinetics curves of the sample at 1000 °C after pre-oxidation at different temperatures for 1 h. The oxidation mass gain trend of the four groups of samples rapidly increases from 0 to 10 h, and the mass increase is slow and relatively stable after 10 h. The sample pre-oxidized at 1000 °C has a trend of rapid mass gain again after 200 h. Compared with the Al-Si coating without pre-oxidation treatment, the oxidation mass gain of the coating sample with pre-oxidation treatment is significantly reduced. The oxidation mass gain of the coating sample with a pre-oxidation temperature of 950 °C is the lowest. At 100 h, the oxidation mass gain of the Al-Si coating is 0.752 mg/cm^2^ at 950 °C, 0.998 mg/cm^2^ at 900 °C, 1.0252 mg/cm^2^ at 1000 °C and 1.521 mg/cm^2^ for the untreated Al-Si coating. This indicates that the pre-oxidation treatment can improve the high-temperature oxidation resistance of the coating and reduce the oxidation loss of Al-Si coating.

**Figure 22:**
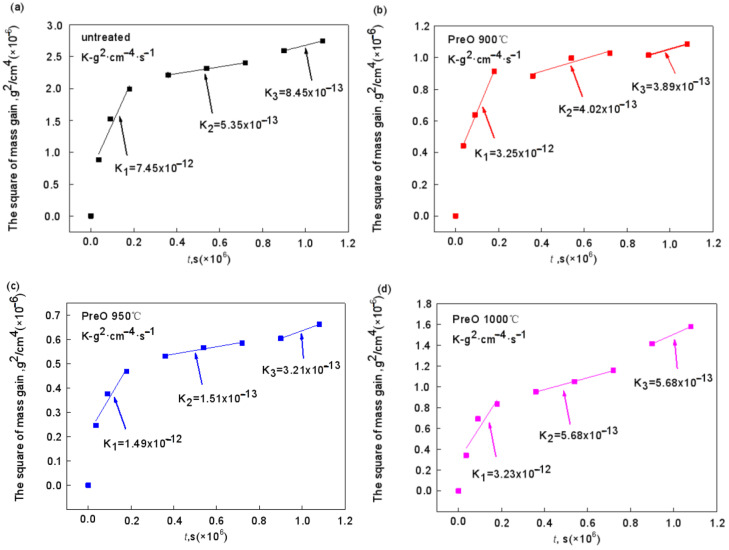
Parabolic rate constant curves of Al-Si coatings under different pre-oxidation temperatures at 1000 °C: (**a**) untreated; (**b**) 900 °C; (**c**) 950 °C; (**d**) 1000 °C.

Combined with Figure 21 and Figure 22, the oxidation trend can be divided into three stages. In the early oxidation stage (0–10 h), the mass gain of oxidation is relatively rapid. The parabolic rate constants of samples without pre-oxidation treatment and 950 °C pre-oxidation treatment are 7.45 × 10^−12^ g^2^·cm^−4^·s^−1^ and 1.49 × 10^−12^ g^2^·cm^−4^·s^−1^, respectively, while those of 900 °C and 1000 °C are almost the same, 3.25 × 10^−12^ g^2^·cm^−4^·s^−1^ and 3.23 × 10^−12^ g^2^·cm^−4^·s^−1^, respectively. At 950 °C, the rate of early mass gain decreased significantly. After oxidation for 10 h, the oxidation rate at 900 °C and 950 °C is relatively stable, and the parabolic rate constant has a small change range, while the sample treated at 1000 °C is almost equal to the sample without pre-oxidation. Within 10–200 h, the parabolic rate constants of the two groups are 5.68 × 10^−13^ g^2^·cm^−4^·s^−^^1^ and 5.35 × 10^−13^ g^2^·cm^−4^·s^−1^, respectively. After 200 h, the mass gain rate at 1000 °C is higher than that without pre-oxidation treatment. The parabolic rate constant reaches 9.14 × 10^−13^ g^2^·cm^−4^·s^−1^, which may be because the element interdiffusion rate between the coating and the matrix is accelerated with the increase in temperature, and the Al consumption is also accelerated. As a result, stable α-Al_2_O_3_ oxide film cannot be formed, and the high-temperature oxidation resistance of the coating is reduced. On the other hand, when the pre-oxidation is at 1000 °C, Cr_2_O_3_ decomposes on the coating surface before high-temperature oxidation, which leads to a thin oxide film on the coating surface. At the same time, the thermal expansion coefficient between the oxide films is quite different such that the surface oxide film is damaged in the high-temperature oxidation stage.

### 4.2. Effect of Pre-Oxidation Time on High-Temperature Oxidation Behavior of Al-Si Coating

**Figure 23:**
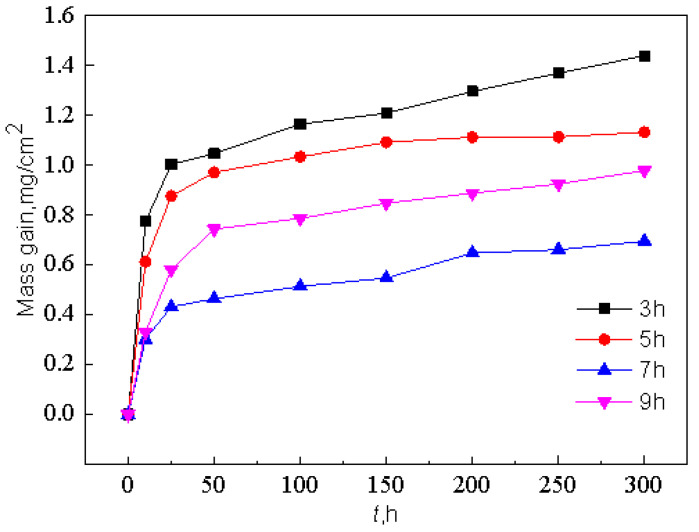
Kinetic curves of constant temperature oxidation of Al-Si coating at 1000 °C under different pre-oxidation times.

**Figure 24:**
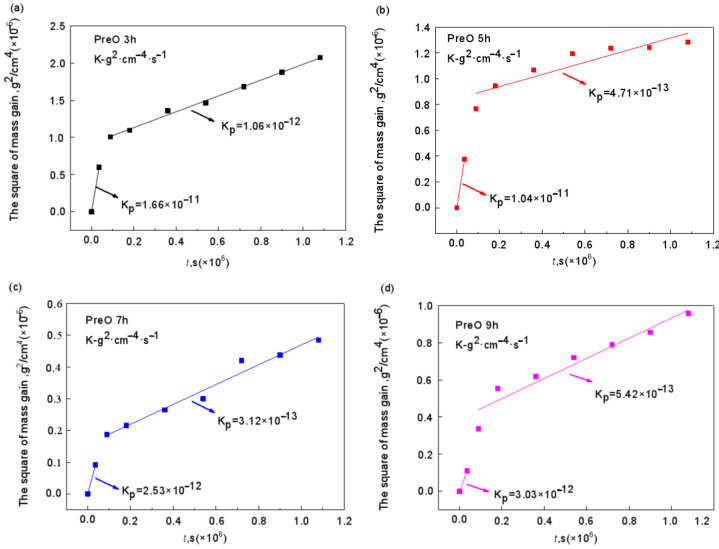
Parabolic rate constant of constant temperature oxidation of Al-Si coating at 1000 °C for 300 h with different pre-oxidation times: (**a**) 3 h; (**b**) 5 h; (**c**) 7 h; (**d**) 9 h.

Figure 23 shows the kinetic curves of constant temperature oxidation at 1000 °C for different pre-oxidation times at 950 °C. With the increase in pre-oxidation time, the oxidation mass gain of Al-Si coating decreased first and then increased, and reached the lowest point at 7 h. Combined with the parabolic rate constant in Figure 24, it can be observed that the pre-oxidation treatment has an obvious effect on the incubation period (0–10 h) of high-temperature oxidation of the coating, and increasing the pre-oxidation time can reduce the oxidation rate of the coating in the early oxidation stage. The pre-oxidation rates of 7 h and 9 h are lower than those of 3 h and 5 h, which are 2.52 × 10^−^^12^ g^2^·cm^−^^4^·s^−^^1^ and 3.03 × 10^−^^12^ g^2^·cm^−^^4^·s^−^^1^, respectively. The oxidation rate constant of the samples pre-oxidized at 7 h is the lowest and remained stable after 10 h of oxidation. After oxidation for 10 h, the parabolic rate constants of the four groups of samples were 1.06 × 10^−^^12^ g^2^·cm^−^^4^·s^−^^1^, 4.71 × 10^−^^13^ g^2^·cm^−^^4^·s^−^^1^, 3.12 × 10^−^^13^ g^2^·cm^−^^4^·s^−^^1^ and 5.42 × 10^−^^13^ g^2^·cm^−^^4^·s^−^^1^, respectively. The mass gain of pre-oxidation 9 h is lower than that of pre-oxidation 5 h, but its parabolic rate constant is larger than that of pre-oxidation 5 h. Therefore, under the same time, the oxidation loss of pre-oxidation 9 h is more serious. Combined with Figure 12 and the oxidation kinetics analysis, it is evident that a relatively complete oxide film has been formed on the surface after 7 h of pre-oxidation. To some extent, increasing the pre-oxidation time can promote further formation of oxide film. At the same time, interdiffusion of elements between the coating and matrix is promoted, and the consumption of Al is increased, which leads to a reduction in the oxidation resistance of the Al-Si coating. In conclusion, 7 h is the appropriate pre-oxidation reference time, and the coating has the best high-temperature oxidation resistance at this time.

### 4.3. Mechanism of Pre-Oxidation on High-Temperature Oxidation Behavior of Al-Si Coatings

It has previously been ascertained that the oxidation mechanism of the Al-Si coating in the high-temperature oxidation process is the continuous destruction and repair of the coating oxide film, that is, the continuous consumption of Al elements in β-NiAl. When the consumption of Al reaches the critical value, it is not enough to generate relatively stable and continuous dense α-Al_2_O_3_ oxide film, and the coating begins to degenerate and flake off, resulting in the failure of the coating. In the process of high-temperature oxidation of the coating, the formation of the oxide film is related to the selective oxidation of the elements with strong oxygen affinity in the coating. Among them, Al will preferentially form the Al_2_O_3_ film due to selective oxidation, and TiO_2_ and Cr_2_O_3_ formed by Ti and Cr will also form the oxide film, which prevents the oxygen ions outside the coating from entering the interior of the coating. In the initial stage of oxidation formation, θ-Al_2_O_3_ is formed, which is unstable and has some defects, such as layer faults and twins. Studies have found that the layer faults interface and twin interfaces easily provide channels for diffusion of Al^3+^ [29,30], which promotes diffusion of Al in the coating and leads to accelerated consumption of Al. When the temperature exceeds 1000 °C, θ-Al_2_O_3_ will transform into α-Al_2_O_3_, which is a stable oxide. The role of pre-oxidation is to promote formation of Al_2_O_3_ in the early oxidation stage of the coating and promote transformation of more θ-Al_2_O_3_ to α-Al_2_O_3_ so as to ensure that more stable α-Al_2_O_3_ oxide film has been generated on the surface of the coating in the early oxidation stage. Increasing the temperature and time of pre-oxidation can promote interdiffusion of elements between the coating and matrix. In the pre-oxidation process, the continuous dense oxide film formed at 950 °C and 7 h prevents the external oxygen ions from entering the matrix. At the same time, formation of M_6_C is promoted at the junction with the matrix, which hinders the mutual diffusion of elements between the matrix and the coating and also reduces diffusion of more Al elements into the matrix. When the temperature exceeds 900 °C, Cr_2_O_3_ easily combines with oxygen to form volatile CrO_3_, which leads to thinning of the oxide film and a reduction in antioxidant performance. However, it is Al_2_O_3_ that really plays the role of oxidation resistance in the coating, and the Al_2_O_3_ film has better oxidation resistance and a lower oxidation rate compared to Cr_2_O_3_ film. Therefore, it is the key to reducing the consumption of Al in the coating and the continuous density of the surface oxide film. Pre-oxidation reduces the Al consumption of the coating in the pre-oxidation stage to a certain extent, and the continuous dense oxide film preformed on the surface reduces the overall oxidation mass gain of the coating, which significantly improves the oxidation resistance and service life of the coating. In addition, it is also found in this experimental study that the pre-oxidized Al-Si-coated samples can still maintain a continuous and intact α-Al_2_O_3_ film under a long duration of high-temperature oxidation.

## 5. Conclusions

In this paper, the effects of pre-oxidation temperature and time on the high-temperature oxidation resistance of Al-Si coating were analyzed. The mechanism of pre-oxidation in the oxidation process of Al-Si coating was analyzed. A process system that can improve the high-temperature oxidation resistance of Al-Si coating was obtained. The specific conclusions are as follows:

(1) A layer of α-Al_2_O_3_ and θ-Al_2_O_3_ oxidation film is formed on the surface of the pre-oxidized Al-Si coating. Within a certain range of pre-oxidation temperature, with an increase in the pre-oxidation temperature, the oxidation mass gain of the coating decreases continuously, and the high-temperature oxidation resistance increases. After 300 h of oxidation, the oxide film on the surface is still continuous and dense, and no peeling phenomenon occurs. When the pre-oxidation temperature is 1000 °C, the decomposition of Cr_2_O_3_ is accelerated, which leads to thinning of the oxide film, an increase in the internal stress between the oxide films and a decrease in the high-temperature oxidation resistance, but it is still higher than that of the Al-Si coating without pre-oxidation.
(2) Under constant temperature oxidation at 1000 °C, the Al-Si coating pre-oxidized at 950 °C for 7 h has the best high-temperature oxidation resistance. Pre-oxidation for 7 h can promote formation of more α-Al_2_O_3_ oxides on the coating surface. After 300 h of oxidation, the oxide film on the coating surface still remains relatively continuous and dense. After 9 h of pre-oxidation, the oxidation mass gain of the coating is also significantly reduced, but, at the same time, the element diffusion between the coating and matrix and the consumption of Al elements are accelerated. After 300 h of oxidation, NiAl_2_O_4_ spinel oxide with poor oxidation resistance is generated on the surface of the coating, resulting in a reduction in the high-temperature oxidation resistance of the coating.
(3) The pre-oxidation process can promote transformation of θ-Al_2_O_3_ to α-Al_2_O_3_ and reduce the effect of the transition of metastable θ-Al_2_O_3_ to α-Al_2_O_3_ in the early oxidation stage. After the pre-oxidation treatment of Al-Si coating, the formation of Si-rich M_6_C prevents interdiffusion of the elements between the substrate and coating and reduces the large consumption of Al in the oxidation process so as to ensure the continuous and dense oxide film formed.

## Figures and Tables

**Table 1:** DZ417G nickel-based alloy composition (wt.%).

Co	Cr	Mo	Al	Ti	V	C	Ni
9–11	8.5–9.5	2.5–3.5	4.8–5.7	4.1–4.7	0.6–0.9	0.11–0.20	Bal.

## Data Availability

Not applicable.
